# Antitumor Activity of New Olivacine Derivatives

**DOI:** 10.3390/molecules25112512

**Published:** 2020-05-28

**Authors:** Janusz Piasny, Benita Wiatrak, Agnieszka Dobosz, Beata Tylińska, Tomasz Gębarowski

**Affiliations:** 1 Department of Basic Medical Sciences, Wroclaw Medical University, 50-556 Wroclaw, Poland; janusz.piasny@student.umed.wroc.pl (J.P.); agnieszka.dobosz@umed.wroc.pl (A.D.); tomasz.gebarowski@umed.wroc.pl (T.G.); 2 Department of Organic Chemistry, Wroclaw Medical University, 50-556 Wroclaw, Poland; beata.tylinska@umed.wroc.pl

**Keywords:** pyridocarbazole, olivacine, P-glycoprotein, multiple drug resistance

## Abstract

Olivacine is an alkaloid-containing pyridocarbazole structure. It is isolated from the bark of the evergreen timber tree, *Aspidosperma olivaceum.* Its well-documented anticancer activity led to the synthesis of new derivatives, which are semisynthetic and fully synthetic pyridocarbazoles. This study aimed to evaluate the potential antineoplastic activity of four newly synthesized olivacine derivatives. Multidrug resistance is a common phenomenon causing failure in the chemotherapy of many tumors. It is mainly related to increased function of P-glycoprotein, an efflux pump removing cytostatic out of the cells. The cell lines used in the study were colorectal carcinoma cell lines: LoVo (doxorubicin-sensitive) and LoVo/DX (doxorubicin-resistant). The NHDF cell line was used to assess cell viability. First, the cells were incubated with olivacine derivatives. In the next step, the following assays were performed: DCF-DA assay, MTT assay, rhodamine 123 assay, detection of apoptosis, proliferation inhibition-mitotic index. The tested compounds showed higher antineoplastic potential and lower toxicity than the reference compound ellipticine. The results indicate that the new olivacine derivatives are good candidates for future anticancer drugs.

## 1. Introduction

Chemotherapy is one of the most common methods of systemic treatment of neoplasms used nowadays in therapy. It is applied either in monotherapy or more often in polytherapy because it is quite effective in reducing tumor mass and growth in many types of neoplasms. Chemotherapy is also combined with other local methods of treatment, including removing/reducing tumor mass with surgery or radiotherapy [1].

To evaluate the efficacy of scheduled therapy in particular clinical cases, one can make in vitro sensitivity assays using isolated cancer cells (from a biopsy or surgical removal of the tumor) [1].

On the other hand, when considering new substances with antineoplastic potential, screening examinations on well-defined cell lines (e.g., tumors derived from ovaries, brain, intestine, lungs, kidneys, blood (leukemia), skin (melanoma), prostate) are carried out [2].

Pyridocarbazoles are a group of well-examined alkaloids with proven anticancer activity. One of their representatives is ellipticine (5,11-dimethyl-6*H*-pyrido [4,3-*b*]carbazole), discovered in the 1960s. It can be found in nature, in the trees of different genera such as *Rauvolfia*, *Ochrosia*, *Aspidosperma*, and *Apocynaceae*. Ellipticine and its derivatives may act in many ways. The main mechanisms are intercalation, inhibition of topoisomerase II, disruption of the cell cycle by interaction with p53 protein, and downregulation of P-glycoprotein (P-gp) expression [3,4,5,6,7,8,9].

Olivacine (1,5-dimethyl-6*H*-pyrido[4,3-*b*]carbazole) is a natural alkaloid, an isomer of ellipticine, first isolated in 1958, from the bark of *Aspidosperma olivaceum*—a Brazilian evergreen timber tree [10,11]. Alkaloids present in the trees of the genus *Aspidosperma* have analgesic, antipyretic, and antibacterial properties. *Aspidosperma* bark extracts are used in traditional medicine to treat malaria. The structural similarity of olivacine and ellipticine also results in them having similar properties [10,11]. It was also noted that some olivacine derivatives show a wider anticancer spectrum and more significant activity compared to ellipticine derivatives and doxorubicin [12,13].

**Figure 1:**
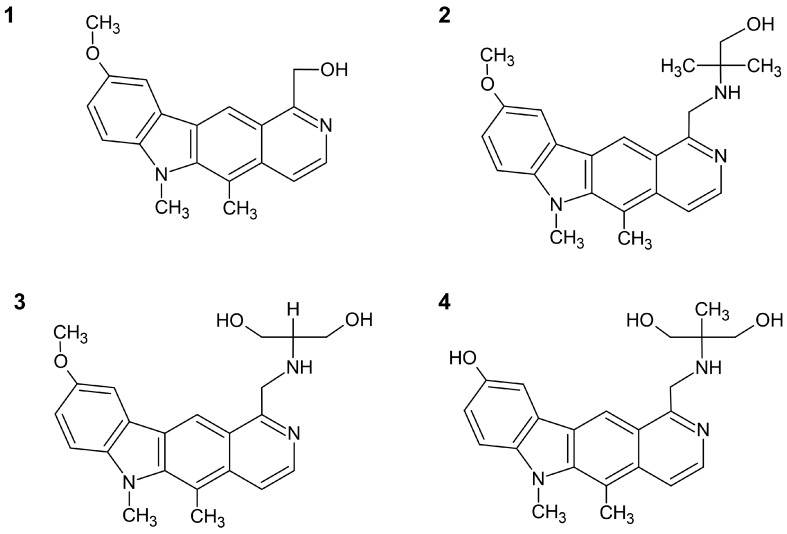
Chemical formulas of tested compounds: (**1**) 5,6-dimethyl-9-methoxy-1-hydroxymethyl-6*H*-pyrido[4,3-*b*]carbazole; (**2**) 5,6-dimethyl-9-methoxy-1-[(1,1-dimethyl-2-hydroxy-ethyl)aminomethyl]-6*H*-pyrido[4,3-*b*] carbazole; (**3**) 5,6-dimethyl-9-methoxy-1-[(1,1-bis-hydroxymethyl-methyl)aminomethyl)]-6*H*-pyrido[4,3-*b*] carbazole; (**4**) 5,6-dimethyl-9-hydroxy-1-[(1,1-bis-hydroxymethyl-ethyl)aminomethyl]-6*H*-pyrido[4,3-*b*] carbazole.

Four new pyrido[4,3-*b*]carbazoles included in the investigation were prepared according to a procedure described previously [14]. They are new olivacine derivatives, modified in positions 1 and 9 of the pyridocarbazole ring. Three new compounds, named **2**, **3**, and **4**, possess alkylamino moieties at the 1-position of 6*H*-pyrido[4,3-*b*]carbazole, while compound **1** is substituted by a methyl hydroxy group (Figure 1). Compounds **1**, **2**, and **3** have a methoxy group in the 9-position of the pyridocarbazole system, while compound **4** has a hydroxy group. All compounds were methylated at position 6.

The series of compounds with alkylamino and methylhydroxy moieties at the 1-position of 6*H*-pyrido[4,3-*b*]carbazole ring system were tested for anticancer activity.

## 2. Results and Discussion

### 2.1. Chemistry

**Figure 2:**
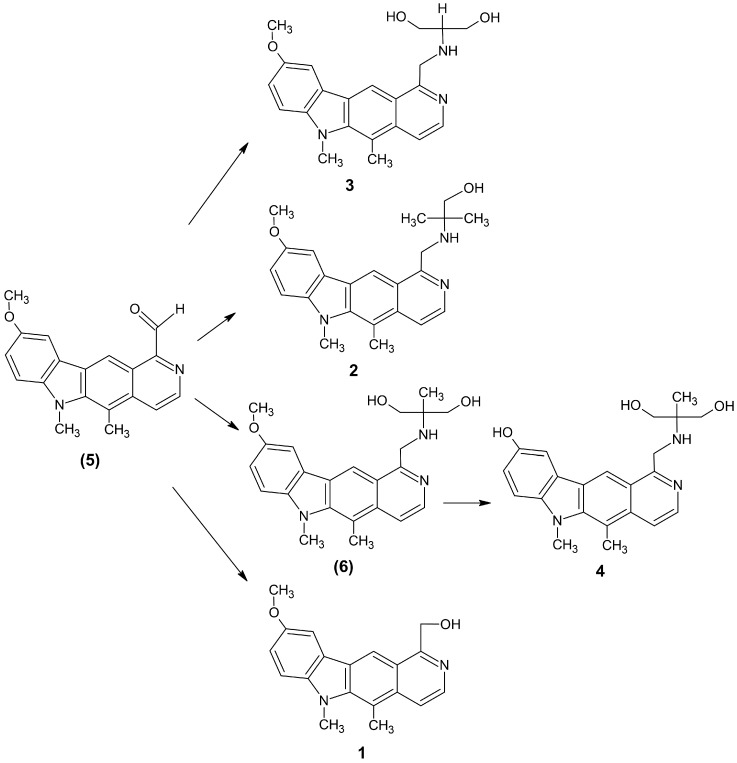
Synthesis scheme of examined olivacine derivatives.

The four olivacine derivatives were synthesized according to a known procedure. Figure 2 presents the synthesis of 1-substituted pyridocarbazole derivatives.

5,6-Dimethyl-9-methoxy-1-[(1,1-bis-hydroxymethyl-methyl)aminomethyl)]-6*H*-pyrido[4,3-*b*]carbazole (**3**) and 5,6-dimethyl-9-methoxy-1-[(1,1-dimethyl-2-hydroxy-ethyl)aminomethyl]-6*H*- pyrido[4,3-*b*]carbazole (**2**) were obtained in the reaction of 5,6-dimethyl-1-formyl-9-methoxy-6*H*- pyrido[4,3-*b*]carbazole (**5**) with 2-amino-1,3-propanediol or 2-amino-2-methyl-1-propanol in toluene. Then the C=N double bond was reduced with sodium borohydride in ethanol. The compounds were obtained in yields ranging from 40% to 48%. The next compound (**6**) was obtained in two steps. 5,6-Dimethyl-9-methoxy-1-[(1,1-bis-hydroxymethyl-ethyl)aminomethyl]-6*H*-pyrido[4,3-*b*]carbazole (**6**) was synthesized according to a similar procedure as that used for compounds **3** and **2**. The resulting pyridocarbazole derivative (**6**) was dissolved in methylene chloride, chilled to −70 °C and boron tribromide was added. The yield of the obtained compound was 35%. 1-Formylpyridocarbazole (**5**) was reacted with sodium borohydride in methanol: chloroform mixture at room temperature for 2 h, giving 5,6-dimethyl-9-methoxy-1-hydroxymethyl-6*H*-pyrido [4,3-*b*]carbazole (**1**), yield 82%. Progress of reactions was observed on TLC.

### 2.2. Biological Evaluation

In this study, the activity of four new olivacine derivatives towards cell viability was examined. The tested cell lines were: NHDF (normal human dermal fibroblasts) and LoVo (human colorectal cancer). The research was carried out using both colorectal cancer cell variations, doxorubicin-sensitive (LoVo) and doxorubicin-resistant (LoVo/DX). The LoVo cell line was purchased from ATCC. At the same time, the resistant version was obtained by incubating the LoVo cell line in a low concentration of doxorubicin for one month (37 °C, 5% CO_2_, 95% humidity), cultured twice a week. This protocol of inducing the resistance of the LoVo cell line was performed and confirmed by Moreira et al. [15]. Since olivacine is not commercially available for research purposes, ellipticine was used as a reference structure.

The MTT assay demonstrated that all examined compounds had a cytotoxicity activity that was directly proportional to the concentration in all cell lines used. To evaluate the concentration over which described derivatives would be cytotoxic for nontumor cells, the NHDF cell line was used.

According to the results, the IC_50_ value was determined (Table 1). IC_50_ allows estimation of the potency of examined substances in inhibiting specific biological functions of cells. All four derivatives showed lower toxicity on NHDF than ellipticine itself (higher IC_50_ value). Simultaneously all compounds had a more toxic effect on the LoVo cell line in comparison to LoVo/DX. The most significant toxic effects were observed after the 24 h incubation, combined with the least harmful impact on NHDF for compound **1**. Among all tested derivatives, compound **1** presented the most relevant cytotoxicity for LoVo and LoVo/DX. Surprisingly, the derivative **3** showed the lowest cytotoxic activity on both LoVo lines and the highest toxicity on NHDF of all the tested compounds.

MTT assay allows evaluating the viability and the cytotoxicity of tested compounds. To estimate if examined derivatives have either cytotoxic or cytostatic mechanism of action, additional assays were performed: proliferation inhibition-mitotic index and detection of apoptosis.

Mitotic index (MI) is a ratio between cells undergoing mitosis to a total number of cells. The proliferation inhibition assay determines the effect of selected compounds on the growth of tumor cells (proliferation) in cultures (LoVo, LoVo/DX, NHDF) indirectly. The slide preparations were made according to the methodology given in the materials and methods section analyzed in a light microscope, counting the percentage of metaphases per 1000 randomly encountered cells in a microscopic image.

According to the results, compound **1** displayed a 50% decline of the mitotic index at 2.3 µM in LoVo and 4.2 µM in LoVo/DX (Table 2). When compared to ellipticine, these values are lower, which may suggest that modification at the 1-position (hydroxyl methyl moiety) determines the beneficial cytostatic effect. Simultaneously, compound **1** had a similar impact on MI in NHDF to ellipticine. Compound **4** showed the lowest IG_50_, but displayed significant proliferation inhibition in NHDF (18.9 µM). The two remaining derivatives, **2** and **3**, impacted MI to a lesser extent than ellipticine, with the least toxic effect in NHDF.

Collaterally, the detection of apoptosis was performed. After incubation with the tested compounds (4 h and 18 h), the cultured cells were stained with a mixture of fluorochromes Annexin V–FITC and propidium iodide and analyzed in a fluorescent microscope. All tested compounds resulted in being inactive at all concentrations and in both incubation periods for both colorectal carcinoma cell lines and the NHDF cell line. Compounds displaying an insignificant impact on the amount of apoptotic or necrotic cells (necrosis growth > 20%) compared to NHDF culture without tested compounds were considered “inactive”. These results may indicate a cytostatic, P-gp, and ROS-dependent mechanism of action of the tested compounds. Since the compounds showed antineoplastic activity in the doxorubicin-resistant LoVo variant, the Rh-123 and DCF-DA assays were performed in the next stage to evaluate their purposefulness in multidrug resistance (MDR).

MDR is one of the significant reasons why tumor transformed cells do not respond to cytostatic treatment. It arises due to increased expression and highlights the function of transporting cell membrane proteins of tumor cells, including P-glycoprotein (P-gp) and ATP-binding cassette, which remove cytostatics out of the cells (an ATP-dependent transport). These proteins act as a sort of self-preserving mechanism of cells against harmful substances and are physiologically present in many tissues, e.g., brain, vessels, endothelium. According to the papers concerning ellipticine—P-gp dependence, some derivatives inhibited P-gp expression while others upregulated it [16].

It seems to be interesting that not only the type of derivative used, but also the oxygen pressure in the tumor cell microenvironment may result in a different expression of P-gp.

**Figure 3:**
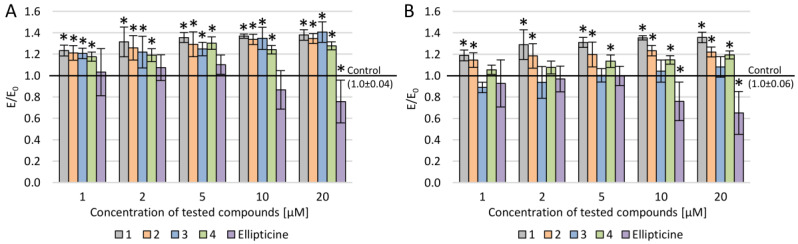
Rhodamine accumulation after incubation with ellipticine and new olivacine derivatives in various concentration ranges: (**A**) LoVo cells, (**B**) LoVo/DX cells; * *p* < 0.05—significant difference compared to control.

P-glycoprotein activity in the presence of newly synthesized compounds was investigated (Figure 3). Rh-123 accumulation assay was performed to evaluate P-gp activity. Jouan et al. examined sixteen substances with P-gp inhibiting potency [17]. Fourteen of them displayed a significant effect on rhodamine 123 accumulation, which is a P-gp substrate. Since the study showed high sensitivity, ca. 80%, the Food and Drug Administration (FDA) approved the method for P-gp activity evaluation. However, P-glycoprotein is not the only protein capable of removing rhodamine 123 out of the cell among the membrane transporters. Therefore, the lack of ABCB1 expression analyses is a limitation of this paper, which we plan to carry out in the next stage of the study.

Rh-123 accumulation was observed in all examined compounds regardless of the used concentration, except compound **3**, where accumulation was observed only for concentrations in the range of 10–20 µM (Figure 3). The Rh-123 accumulation in LoVo was slightly higher than in LoVo/DX in all cases. Furthermore, ellipticine did not show any impact on Rh-123 accumulation in LoVo/DX, and its effect in LoVo was observed only in a narrow concentration range (1–5 µM), where accumulation was lower than 6%. Palko-Labuz et al. demonstrated stronger overexpression of multidrug transporter P-gp in LoVo/DX in comparison to LoVo [18].

Despite this, the new olivacine derivatives, excluding compound **3**, showed significant inhibition of P-gp for the LoVo/DX cell lines. In normal tissues, chronic exposure to an increased ROS level leads to adaptive changes and the initiation of tumorigenesis. Cancer cells achieve an intensive metabolic rate. Therefore, when compared with nontumorous cells, they display higher levels of free radicals. Despite increased intracellular ROS levels, cancer cells remain oxidative-stress-sensitive; ROS exceeding a certain level can kill cancer cells. Cytotoxicity of chemotherapeutics is often related to ROS amplification. However, under chronic exposure to free radicals, cancer cells develop drug resistance (P-gp, transmembrane proteins) [19,20].

According to previous papers, the level of intracellular ROS between drug-resistant cells and their sensitive counterparts differs significantly. Moreira et al. [21] demonstrated that the LoVo/DX cell line displayed a significantly lower level of intracellular ROS than the drug-sensitive line LoVo. Maiti had similar results comparing ovarian cancer cell lines [22].

**Figure 4:**
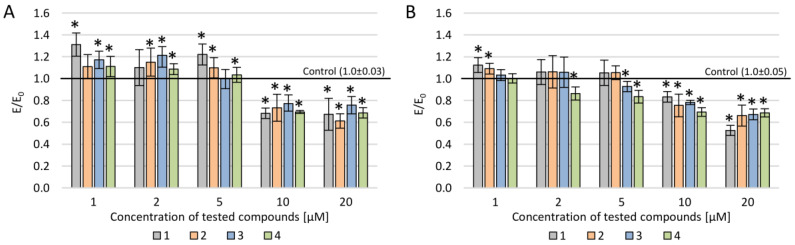
DCF-DA assay—DCF (2′,7′-dichlorofluorescein) concentration after incubation with new olivacine derivatives in various concentration ranges: (**A**) LoVo cells, (**B**) LoVo/DX cells; * *p* < 0.05—significant difference compared to control.

The DCF-DA assay showed that all four new olivacine derivatives demonstrated prooxidative activity, which was observed at a low concentration range (1–5 µM) for LoVo and LoVo/DX cell lines (Figure 4). In contrast, when incubated at a higher concentration range (10–20 µM), the tested compounds displayed antiradical activity. Concerning the prooxidative potency, derivative **1** showed the strongest activity in the 1–5 µM concentration range for both LoVo lines. In LoVo/DX, the significant prooxidative effect was also observed for compound **2** at the lowest concentration (1 µM). Both derivatives share the same moiety at the 9-position (methoxy) what may suggest that substitution with methoxy group at this position affects prooxidative activity in LoVo/DX. ROS-induced DNA damage is a well-known mechanism of action of many cytostatics [23]. Elevating the ROS level over a certain level may be an important factor in tumor chemotherapy since the initial level of intracellular ROS level for LoVo/DX is lower than for LoVo [21,22].

Examination of DU-145 cancer cell lines (prostate cancer) demonstrated a correlation between HIF-1 alpha, ROS level, and P-gp expression. While the reactive oxygen species level decreased, the HIF-1 alpha increased, which resulted in the upregulation of P-gp (under physiological and chemically induced hypoxia). On the other hand, the small increase in cellular ROS level (usage of H_2_O_2_) downregulated the expression of P-gp and HIF-1 alpha. To confirm the result, cell lines were coincubated with free radical scavengers, and the results were similar to those obtained under hypoxic conditions [24].

Considering the possibility of MDR abolishment due to keeping ROS in the tumor environment at the proper level is exciting and important in the new approach to the effectiveness of the therapy.

The rhodamine accumulation assay (Rh-123) is used to estimate P-gp transporting activity in cultured cells after incubation with chemical compounds changing their expression. The intracellular growth of Rh-123 allows decreased P-gp activity to be measured.

For all newly examined derivatives, rhodamine accumulation was observed, but only for compound **1** in the full range of concentration (1–20 μM).

Due to many toxicity-related adverse effects (nephrotoxic activity, immunohemolytic effect) and low bioavailability and solubility, ellipticine and its analogs were withdrawn. However, promising anticancer activities of the ellipticine isomer olivacine and its analogs have resulted in many papers and further research.

Compound **1** has the least complex structure in comparison to the other three examined olivacine derivatives. It is the only compound that has a methylhydroxy moiety at the 1-position, whereas the rest of the derivatives used in this study have alkylamino moieties at this position. Both modifications at 1-position improve hydrophilic properties of derivatives in comparison to the ellipticine, so the solubility in water is better [25]. Substitution at the 9-position seems to be a crucial factor for prooxidative activity since compounds with a methoxy group (**1**, **2**, and **3**) at this position displayed higher intracellular ROS levels when incubated with colorectal cancer cells than compound **4**, which has a hydroxy group.

Pyridocarbazoles are a group characterized by an omnidirectional mechanism of action [4,5,6,7,8,9]. Hence, the interaction with DNA and the way the new derivatives influence p53 protein function have to be explored in the future.

The ellipticine molecule’s size and shape are similar to purine–pyrimidine pairs, which enables ellipticine to intercalate between DNA helix chains effectively. Furthermore, as a polycyclic molecule, it can reach hydrophobic regions of DNA. Interactions of ellipticine and thymine methyl groups are useful in predicting where the binding region of the compound is [26,27].

Inhibiting topoisomerase II with ellipticine is a well-documented mechanism of action [4,5,6,9]. Ellipticine may act by binding to the enzyme in the absence of nucleic acid, as well as by binding to the topo II–DNA complex [8,9]. Forming the topo II–DNA complex increases the number of DNA ruptures, which leads to the death of the cell.

In over 55% of carcinoma cases, the loss of p53 function is observed. p53 is an important transcription factor for many proteins, including proliferation-inhibiting proteins and proteins that trigger apoptosis in the damaged cells. The main reasons for the impaired functions are point mutations in the TP53 gene [7,28,29,30,31].

The MDM2 protein is responsible for regulating the p53 level in cells; it has ubiquitin ligase activity and binds specifically to p53. The dependence between these proteins is based on negative feedback: p53 stimulates transcription of MDM2. Defective p53 loses activity for stimulating cell cycle regulation; it neither induces apoptosis nor regulates its negative transcription factor MDM2. Thus, cancer cells are characterized by a significant increase of faulty p53 (as a result of reduced degradation due to the shortage of MDM2) [7,31].

Reconstitution of p53 leads to inhibition of the cell cycle (inducing p^21WAF1/CIP1^ transcription) and increases the apoptosis of tumor cells. Moreover, it should also sensitize the tumor cells to cytostatic therapy since the lack of apoptosis-triggering factors from defective p53 is an important reason for drug resistance [29].

Ellipticine has proven activity in restoring the transcription function of p53 [7,28,29,30]. In cell culture incubated with a 9-hydroxy derivative, a significant increase in transcription of p^21WAF1/CIP1^ and Bax has been observed, as well as strongly inhibited proliferation and intensified apoptosis. It is notable that ellipticine affected only the defective fraction of p53. Such an impact was not observed either in normal cells or tumor cells without p53 mutation [7]. This selectivity is a hopeful prospect for preserving unmutated cells.

Similar studies were performed by Gębarowski et al., who examined the p53-restoring potential of three newly synthesized olivacine derivatives [32]. The evaluation was based on multiple-criteria decision analysis, including the rate of apoptosis, p^21Cip1/Waf1^ level, and P-gp inhibition. The study was performed on two tumor lines: CCRF/CEM (contains mutant p53) and A549 (includes wild-type p53). The individual results were then referred to as the intracellular p53 level. Beneficial anticancer activity of these derivatives was observed (including an increase in apoptosis and the number of dead cells, and inhibition of the cell cycle).

Interestingly, the compound that displayed the most significant anticancer activity in the quoted study (restoring the function of both p53 variants, mutant, and wild-type) shared structural features with compound **1** examined in this study. Both derivatives have the same pyridocarbazole main structure, are methylated at the 6-position, and have the methyl hydroxy substitution at the 1-position, which is relatively the least complex moiety in comparison to other reviewed compounds. This correlation may indicate that compound **1** may have the highest potential in p53 reconstitution. Worth mentioning, compound **3** shares a very similar structure with a derivative that reactivated selectively with mutant p53 (pyridocarbazole structure methylated at the 6-position, with a methoxy moiety at the 9-position, and an alkylamino moiety with hydroxy groups at the 1-position).

## 3. Materials and Methods

### 3.1. Tested Compounds

In the present study, the anticancer activity of new olivacine derivatives was examined. The compounds were synthesized in the Department of Organic Chemistry of Wroclaw Medical University. Tested compounds were dissolved in DMSO to reach a final concentration of 10 mM and stored at −20 °C. Briefly, before use, the solutions of the compounds were dissolved in a complete medium. Samples with four different concentrations were prepared for each compound: 1, 2, 5, 10, and 20 μM. At the highest concentration of each compound, the DMSO content did not exceed 0.2%.

### 3.2. Cell Lines and Conditions

The cytotoxicity was evaluated using three cell lines: normal human dermal fibroblasts (NHDF, CC-2511, Lonza, Basel, Switzerland) and two colorectal carcinoma cell lines—LoVo (CCL-229) obtained from ATCC (Manassas, VA, USA) and LoVo/DX (doxorubicin-resistant), which was prepared by incubation of LoVo with a low concentration of doxorubicin for three months. As far as cytotoxicity evaluation is concerned, it needs to be pointed out that the colorectal epithelium cell line would be the most optimal choice for the nontumor cell line. Usage of NHDF instead (it was the only human cell line available at a laboratory at the time when experiments were performed) is a limitation of this paper. Both cancer lines were cultured in complete medium (DMEM F-12 supplemented with 10% FBS, 2 mM L-glutamine, and 25 μg/mL gentamicin) at 5% CO_2_, 37 °C, 95% humidity and subcultured with TrypLE twice a week. The NHDF cell line was grown in DMEM without phenol red with the addition of 10% FBS and 2 mM L-glutamine and 25 μg/mL gentamicin. This cell line was incubated at the same conditions as cancer cells.

### 3.3. DCF-DA Assay

The level of reactive oxygen species (ROS) can be defined by the spectrofluorometric measurement of 2′,7′-dichlorofluorescein (DCF). DCF-DA (2′,7′-dichlorofluorescin diacetate; D6883, Sigma Aldrich, St. Louis, MO, USA) is deacetylated by esterases to a nonfluorescent compound that, in the presence of ROS, is oxidized to DCF. The lower the DCF level is, the smaller is the number of free radicals in the sample. The DCF-DA solution was prepared freshly before use by dissolving 1 mg of DCF-DA in 2.05 mL of 100% ethanol and diluting it in deionized water to a final concentration of 10 μM. H_2_O_2_ (hydrogen peroxide) was used as a source of endogenous stress (100 μM). After 1 h of incubation (96-well plates, 10,000 cells/well) with compounds, the culture medium was removed, and cells were rinsed with PBS. Then, 25 µM of DCF-DA solution in MEM without serum and phenol red was added for 1 h at 37 °C. The level of ROS was measured fluorimetrically with excitation at 485 nm and emission at 535 nm using a Victor2 microplate reader (PerkinElmer, Waltham, MA, USA).

### 3.4. MTT Assay

MTT allows estimation of the viability of cells. After dividing the supernatant, the cells were rinsed with PBS, then tetrazole salt in a concentration of 1 mg/mL was put into each well (96-well plates, 10,000 cells/well). In the next 2 h, the culture was incubated in a CO_2_ incubator at 37 °C, then purple crystals of formazan were dissolved in 100 µL of isopropanol, in the dark, and vortexed/shaken for 30 min. After that, the spectrophotometric measurement was taken at 555 nm using a Victor2 microplate reader.

### 3.5. Accumulation of Rhodamine 123

Rhodamine 123 accumulation assay was performed to determine the effect of tested compounds on P-glycoprotein.

The cells were previously incubated with tested compounds in 96-well plates (10,000 cells/well) for 24 h, then Rh-123 was added (the final concentration was 12.5 μM) and incubated for 60 min. Subsequently, the plates were shaken at 500× *g*, 10 min, and then the supernatant was removed. To dissolve the cells, 20 mM Tris–HCl (pH 7.7) containing 0.2% sodium dodecyl sulfate (SDS) was used, which resulted in lysis of the cells and release of the intracellular fluorescent substrate. Fluorescence was measured with excitation at 485 nm and emission at 535 nm using a Victor2 microplate reader.

### 3.6. Detection of Apoptosis

Cells were cultured in 24-well plates (50,000 cells/well) for 48 h at 37 °C, 5% CO_2_. After this time, the surface of the wells was covered with the cells of adherent line, then the tested compounds were added at concentrations of 1, 2, 5, 10, and 20 μM for 4 or 18 h. Medium from each well was harvested into centrifuge tubes (supernatant may contain nonadherent, dead or apoptotic cells). Then, the wells were washed with a trypsin–EDTA solution, and supernatants were also collected into tubes. In the next step, the cells were treated again with trypsin–EDTA and collected into tubes. The tubes were then centrifuged at 600× *g* for 10 min at 20 °C for 10 min. The cell pellet was suspended in 100 μL of HEPES–NaOH buffer at pH 7.5, and a mixture of fluorochromes Annexin V-FITC and propidium iodide was added and left in the dark for 10 min. The preparations were analyzed using Eclipse E600 microscope (Nikon, Kanagawa, Japan).

### 3.7. Proliferation Inhibition—Mitotic Index

Cells were cultured in 24-well (50,000 cells/well) plates at 37 °C, 5% CO_2_ with tested compounds for 18 h. For the last 4 h of the culture, colcemid solution was added (0.1 μM/mL) to block mitotic pitch spindle. Next, the cells were removed from the surface of the wells, and the suspension was centrifuged at 600× *g* for 10 min at 20 °C for 10 min. The cells were treated with a hypotonic 0.075 M KCl solution, added dropwise to the pellet with careful pipetting. The cells remained suspended in a hypotonic solution at 37 °C for 45 min; then, the suspensions were centrifuged 600× *g* at 20 °C for 10 min. The cells were fixed in a cold (4 °C) mixture of methanol and glacial acetic acid in a 3:1 volume ratio by changing the fixative three times. After each fixation, the cells were centrifuged at 600× *g* at 4 °C for 10 min. After fixation, the cells were suspended in a volume of 100 mL fixative and spotted onto previously prepared defatted and frozen slides. The preparations were left to dry. The next day, the preparations were stained for 15 min in a 20% aqueous solution of Giemsa dye and analyzed in a light microscope (Nikon Eclipse E600, Kanagawa, Japan) at 400 × magnification, assessing the percentage of metaphases calculated per 1000 randomly encountered cells in the microscopic image.

The cell growth inhibition factor (CGIF) was calculated according to Equation (1):

CGIF = 100 − (% metaphases of tested cytostatics dose × 100)/(% metaphases of control)(1)

Then, using the calculated coefficients for each of the tested compounds, the regression equation was calculated from the regression equation what determined the concentration of the tested compound IG_50_ (growth inhibition 50%, i.e., the concentration at which the mitotic index was reduced by 50%).

### 3.8. Statistical Analysis

The normal distribution was checked using the Shapiro–Wilk test, and equality of variances was checked by Levene’s test. ANOVA and Tukey post-hoc tests were performed. The level of statistical significance was assumed to be *p* < 0.05.

## 4. Conclusions

For all tested pyridocarbazoles, the accumulation of rhodamine in the rhodamine 123 assay was observed. Compounds **1**, **2**, and **4** displayed rhodamine accumulation in the full concentration range for LoVo as well as LoVo/DX. The exception was compound **3**, which showed this activity in LoVo/DX only in the 10–20 μM range. The reference compound ellipticine impacted Rh-123 accumulation only in the LoVo cell line in the 1–5 μM range. This may indicate the usefulness of these derivatives as they were more effective in P-gp inhibition and cytostatic activity than ellipticine.

The prooxidative activity was observed for all tested compounds for both LoVo cell lines, notably in the range of 1–5 μM. Compound **1** had the most significant effect of increasing the intracellular ROS level. When analyzing previous papers according to the chemical structure of the compound, one can say that the substitution at the 9-position and the type of used moieties in pyridocarbazoles are the important factors determining prooxidative activity.

According to the results presented in this paper, compound **1** is the most promising molecule for further research in the context of the antineoplastic activity.

## Figures and Tables

**Table 1:** Results of MTT assay—IC_50_ values for tested compounds in various cell lines.

	Cell Line	IC_50_ [µM]		
Compound		LoVo	LoVo/DX	NHDF
**1**		4.84 ± 1.03	16.42 ± 0.49	74.03 ± 5.12
**2**		13.50 ± 0.80	20.03 ± 3.86	58.78 ± 2.14
**3**		15.43 ± 0.26	22.21 ± 0.79	41.08 ± 4.77
**4**		9.37 ± 0.13	19.36 ± 2.43	52.30 ± 2.43
Ellipticine		4.28 ± 0.53	18.16 ± 0.34	22.45 ± 3.14

**Table 2:** Mitotic index evaluation—IG_50_ values for tested compounds in various cell lines.

	Cell Line	Cell Line		
Compound		LoVo	LoVo/DX	NHDF
**1**		2.3 ± 0.9	4.2 ± 0.9	20.3 ± 7.8
**2**		5.8 ± 2.9	6.5 ± 1.9	23.5 ± 12.6
**3**		7.4 ± 2.9	12.5 ± 2.2	21.4 ± 1.6
**4**		3.2 ± 0.7	4.7 ± 1.0	18.9 ± 2.1
Ellipticine		3.4 ± 1.0	7.0 ± 3.7	21.2 ± 7.8
